# Reduced 25-hydroxyvitamin D concentration in the aqueous humor of cataract patients with open-angle glaucoma

**DOI:** 10.1038/s41598-021-98342-9

**Published:** 2021-09-22

**Authors:** Yongwun Cho, Seung Pil Yun, Woong-Sun Yoo, Rock-Bum Kim, Min-Chul Cho, Seong-Jae Kim

**Affiliations:** 1 Department of Ophthalmology, Gyeongsang National University Hospital and Gyeongsang National University College of Medicine, 79 Gangnam-ro (90 Chiram-dong), Jinju-si, Gyeongsangnam-do 52727 Korea; 2 Institute of Health Science, Gyeongsang National University, Jinju, Korea; 3 Department of Preventive Medicine, Gyeongsang National University Hospital and Gyeongsang National University College of Medicine, Jinju, Korea; 4 Department of Pharmacology and Convergence Medical Science, Institute of Health Sciences, College of Medicine, Gyeongsang National University, Jinju, Korea; 5 Department of Laboratory Medicine, Gyeongsang National University Hospital and Gyeongsang National University College of Medicine, Jinju, Korea

**Keywords:** Glaucoma, Risk factors

## Abstract

Previous epidemiological studies have demonstrated that the lower serum concentration of vitamin D was associated with elevated risk of open-angle glaucoma (OAG). However, few studies have examined the association between aqueous humor vitamin D concentrations and OAG. Hence, we investigated the relationship between 25-hydroxyvitamin D (25(OH)D) concentrations in aqueous humor and OAG. We measured 25(OH)D concentrations in aqueous humor and serum of 126 patients who underwent cataract surgery. 36 were patients with OAG and 90 were control patients. The 25(OH)D concentrations were measured using Elecsys Vitamin D Total Kits with the Cobas e602 module (Roche Diagnostics, Mannheim, Germany), an electrochemiluminescence assay. Multiple linear regression analysis was performed to investigate factors associated with serum and aqueous humor 25(OH)D concentrations. Patients with OAG had significantly lower 25(OH)D concentrations in aqueous humor than control patients. Serum 25(OH)D concentrations were higher in patients with OAG than in the control, but this was not statistically significant. 25(OH)D concentrations in aqueous humor of patients with OAG were significantly associated with axial length but not with glaucoma severity, which was determined by the retinal nerve fiber layer thickness or mean deviation. Vitamin D concentrations in aqueous humor of patients with OAG were significantly lower than those in patients without OAG.

## Introduction

Glaucoma is a leading cause of irreversible blindness worldwide, contributing to approximately 10% of cases of legal blindness registered in the United States^1^. The core event in glaucoma is irreversible damage of retinal ganglion cell axons, which carry visual information from the eye to the brain, due to elevated intraocular pressure (IOP)^2^. Depending on the mechanism of increasing IOP, glaucoma could be classified into two categories: angle-closure glaucoma (ACG) or open-angle glaucoma (OAG). Unlike ACG, which has a narrow iridocorneal angle, OAG is characterized by increased resistance in the trabecular meshwork (TM), which causes an elevation of IOP. Several studies have suggested that changes in the concentration of various molecules in aqueous humor, such as vitamin C^3^, hyaluronic acid^4^, transforming growth factor β (TGF-β)^5^, and endothelin-1^6^, increase TM resistance.

Vitamin D does not exhibit biological activity until two-step hydroxylation occurs. Following the hydroxylation of vitamin D into 25-hydroxy vitamin D [25(OH)D] in the liver, the vitamin D metabolite is transported to kidneys where it is converted to 1α, 25-dihydroxyvitamin D [1α, 25(OH)2D], which is an active form of vitamin D^7,8^. The vitamin D status is usually evaluated by measuring 25(OH)D concentrations. Its deficiency affects bone and mineral metabolism; however, recent studies have reported that vitamin D insufficiency is associated with various systemic diseases. Adequate vitamin D intake can prevent diseases, such as myocardial infarction, stroke, diabetes mellitus types 1 and 2, infectious or chronic respiratory diseases, and autoimmune diseases^8–12^. Furthermore, the serum concentrations of vitamin D has been linked to the prevalence or occurrence of eye disorders, such as diabetic retinopathy, age-related macular degeneration, myopia, and dry eye syndrome^13–16^. In particular, several studies have demonstrated that the serum concentration of vitamin D is associated with OAG^17–22^.

However, most of these studies were only epidemiological investigations of the association between serum vitamin D concentrations and eye diseases; few studies have examined the association between aqueous humor vitamin D concentrations and eye diseases. To the best of our knowledge, no studies have reported on the relationship between the aqueous humor vitamin D concentration and glaucoma. In the present study, we measured 25(OH)D concentrations in aqueous humor of patients with cataract with or without OAG. We then performed a comparative analysis of the 25(OH)D concentrations in the two groups to determine whether there was a correlation with the presence of OAG.

## Results

Total 126 patients were enrolled in this study, and 72 were men and 54 were women. The OAG group comprised 18 men and 18 women, and the control group comprised 54 men and 36 women. The duration of outdoor activity that could affect the vitamin D concentrations was 2.83 ± 2.50 h/day in the OAG group and 2.78 ± 2.24 h/day in the control group. The mean ages of patients in the OAG and control groups were 64.36 ± 11.77 and 66.26 ± 14.55 years, respectively. There were no significant differences in the sex, age, mean duration of outdoor activity, severity of cataract, and underlying diseases between the two groups (Table 1). In patients with glaucoma, the mean RNFL thickness measured by optical coherence tomography was 65.81 μm, and the mean deviation value obtained from the visual field test was 16.4 dB. Visual acuity was significantly lower, AL was significantly higher (*p* =  < 0.01), and CCT was significantly lower (*p* = 0.03) in the OAG group than in the control group. In contrast, there was no significant difference between the two groups in terms of IOP, ECC, and ACD (Table 1).

**Table 1:** Demographic and clinical characteristics of patients with open-angle glaucoma and control subjects.

Variables	Total (% or SD)	Control (% or SD)	OAG (% or SD)	*p* value*
Total	126	90	36	0.32
Male	72 (57.14)	54 (60.00)	18 (50.00)	
Female	54 (42.86)	36 (40.00)	18 (50.00)	
Age (years)	65.71 ± 13.79	66.26 ± 14.55	64.36 ± 11.77	0.23
Average outdoor activity time (h/day)	2.80 ± 2.31	2.78 ± 2.24	2.83 ± 2.50	0.98
Mean RNFL thickness			65.81 ± 18.81	
Mean visual field MD			− 16.4 ± 8.67	
Axial length (mm)	23.85 (1.91)	23.63 (1.88)	24.39 (1.89)	< 0.01
ACD (mm)	2.75 (0.61)	2.77 (0.63)	2.71 (0.58)	0.71
AqH vitamin D concentration (ng/mL)	10.06 (5.60)	11.52 (5.77)	6.42 (2.89)	< 0.01
Serum vitamin D concentration (ng/mL)	19.01 (10.42)	18.12 (9.55)	21.22 (12.18)	0.27
BCVA (LogMAR)	0.45 (0.30)	0.39 (0.29)	0.60 (0.28)	< 0.01
IOP (mmHg)	15.84 (5.34)	15.08 (4.71)	17.75 (6.34)	0.10
CCT (µm)	541.2 (36.60)	546.4 (34.78)	528.1 (38.25)	0.03
ECC (/mm^3^)	2463 (530.2)	2446 (535.6)	2504 (522.3)	0.20
Cataract grade (LOCSIII)	2.8 ± 0.90	2.9 ± 0.97	2.7 ± 0.92	0.34

*OAG* open-angle glaucoma, *SD* standard deviation, *RNFL* retinal nerve fiber layer, *MD* mean deviation, *ACD* anterior chamber depth, *AqH* aqueous humor, *BCVA* best-corrected visual acuity, *IOP* intraocular pressure, *CCT* central corneal thickness, *ECC* endothelial cell count, *LOCSIII* lens opacities classification system III.

*Comparisons between patients with OAG and control subjects based on *t*-test.

**Figure 1:**
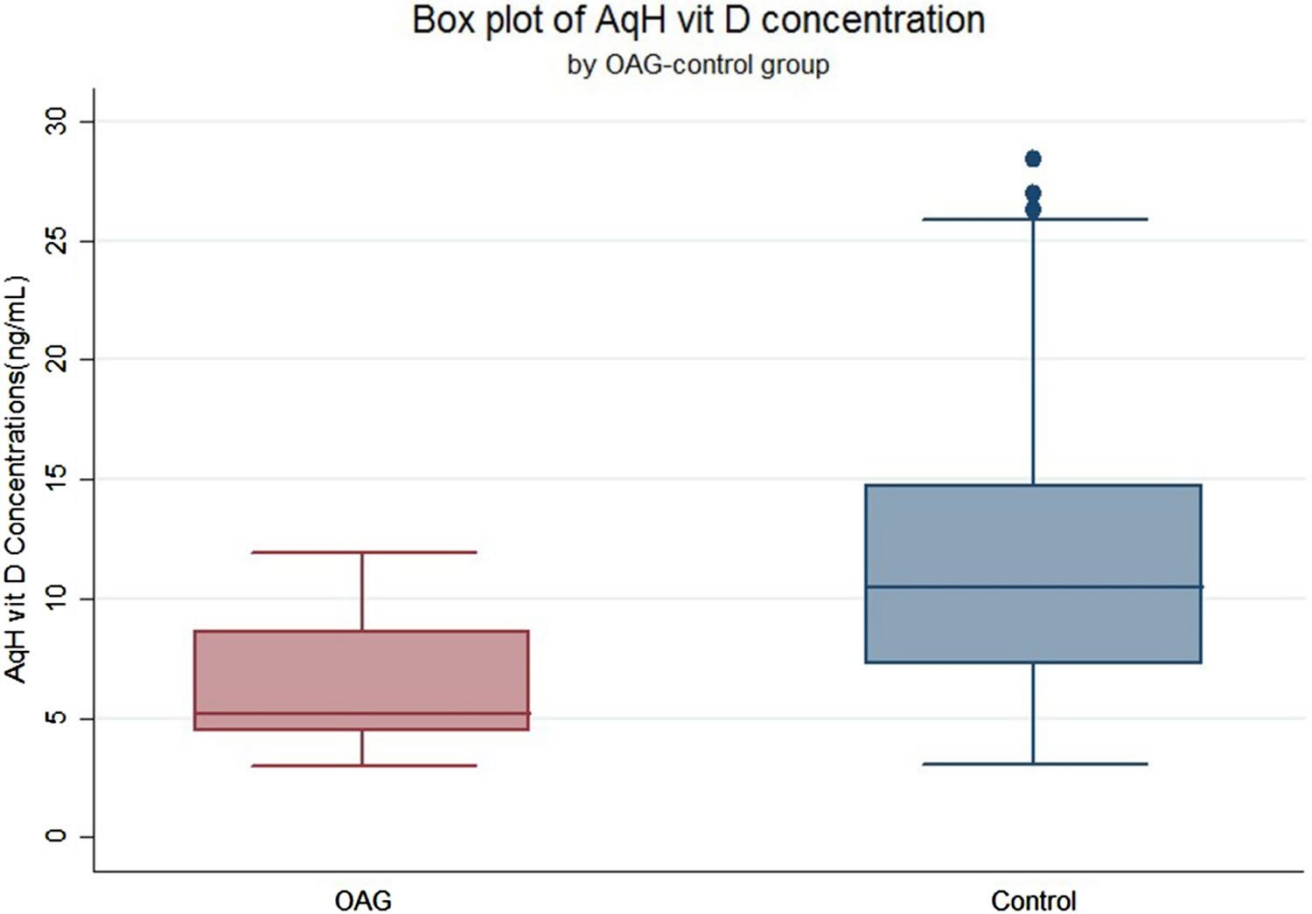
Concentrations of 25(OH)D in the aqueous humor of patients with open-angle glaucoma vs. control subjects. OAG = open-angle glaucoma.

The serum 25(OH)D concentrations were 21.22 ± 12.18 ng/mL in the OAG group and 18.12 ± 9.55 ng/mL in the control group, which was slightly but not significantly higher in the OAG group (Table 1). However, the vitamin D concentrations in aqueous humor were 6.42 ± 2.89 ng/mL in the OAG group and 11.52 ± 5.77 ng/mL in the control group, and the difference between the two groups was statistically significant (*p* < 0.01; Fig. 1; Table 1).

**Figure 2:**
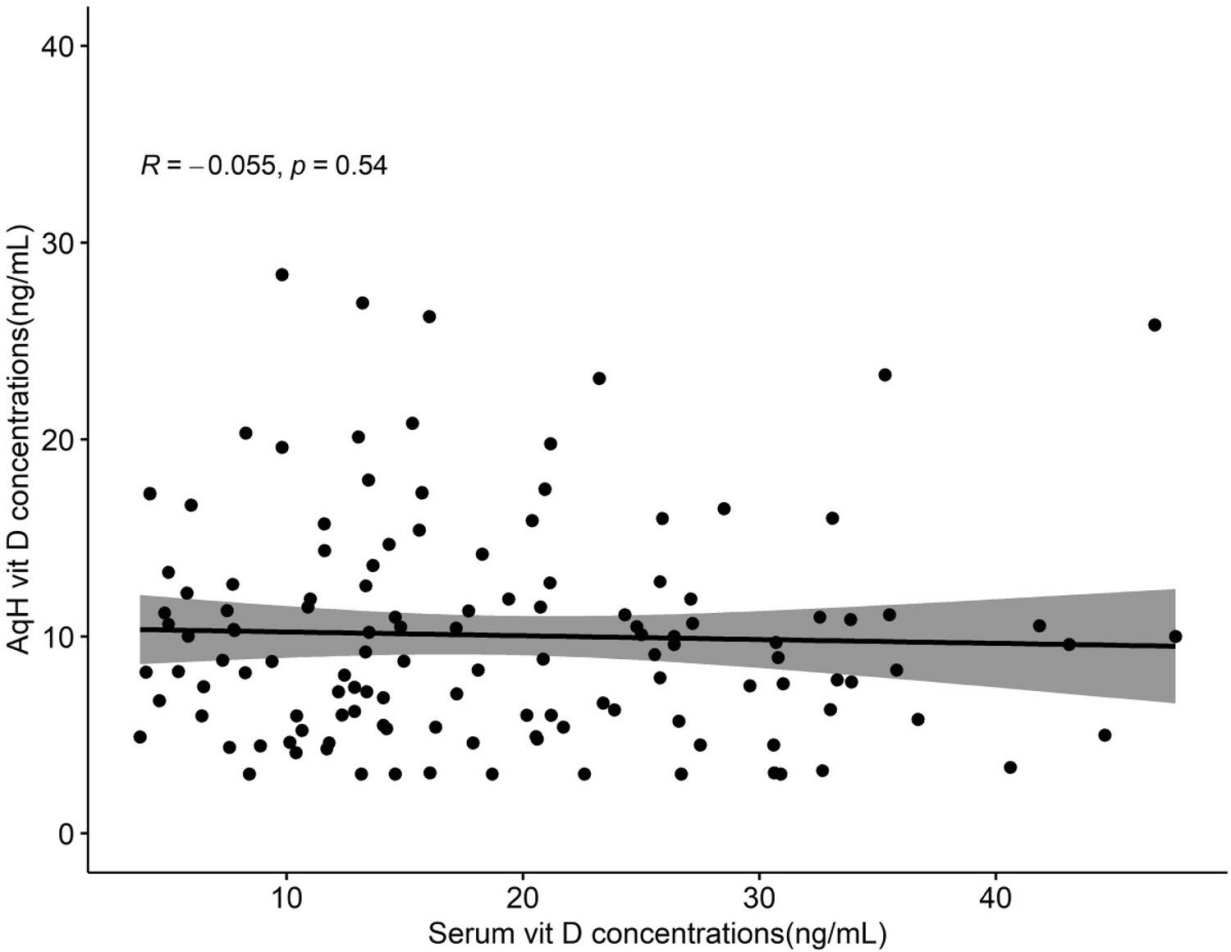
The correlation plot between serum and aqueous 25(OH)D levels of all patients. AqH = aqueous humor; vit D = vitamin D.

A multivariate linear regression analysis targeting 25(OH)D concentrations in aqueous humor was conducted for all patients, and the results showed that the presence of OAG had a significant effect on the 25(OH)D concentration in aqueous humor (odds ratio [OR] 0.57, 95% confidence interval [CI] 0.47–0.69, *p* < 0.01; adjusted OR 0.64, 95% CI 0.53–0.78, *p* < 0.01, Table 2). In the same analysis, the 25(OH)D concentration in aqueous humor was significantly higher for males (OR 1.3, 95% CI 1.07–1.57, *p* =  < 0.01; adjusted OR 1.27, 95% CI 1.06–1.52, *p* =  < 0.01), whereas the concentration became statistically lower as the AL increased (OR 0.96, 95% CI 0.91–1.00, *p* = 0.07; adjusted OR 0.93, 95% CI 0.88–0.99, *p* = 0.01) (Table 2). However, there was no relationship between the 25(OH)D concentration in serum or the spherical equivalent and the concentration in aqueous humor. Figure 2 shows the correlation plot between serum and aqueous 25(OH)D concentrations of all patients.

**Table 2:** Multivariate linear regression analysis of factors associated with 25(OH)D concentrations in the aqueous humor (in all patients).

Variables	OR (95% CI)	*p* value*	Adjusted OR (95% CI)	*p* value**
OAG	0.57 (0.47–0.69)	< 0.000	0.64 (0.53–0.78)	< 0.01
Control	1.00		1.00	
Serum vitamin D concentrations	1.00 (0.99–1.01)	0.557	1.00(0.99–1.00)	0.27
Sex				
Male	1.3 (1.07–1.57)	0.007	1.27 (1.06–1.52)	< 0.01
Female	1.00		1.00	
Age (10 years)	1.00 (0.99–1.01)	0.847		
Average RNFL thickness	1.09 (1.04–1.14)	0.004		
Visual field MD	1.00 (0.98–1.02)	0.836		
IOP	1.01 (0.99–1.02)	0.565		
Spherical equivalent	0.96 (0.93–0.99)	0.019	0.98 (0.96–1.01)	0.28
Axial length	0.96 (0.91–1.00)	0.076	0.93 (0.88–0.99)	0.01
Anterior chamber depth	1.10 (0.94–1.29)	0.225		

*OAG* open-angle glaucoma, *RNFL* retinal nerve fiber layer, *MD* mean deviation, *IOP* intraocular pressure, *CI* confidence interval, *OR* odds ratio.

*Simple linear regression, **multiple linear regression.

Multivariate linear regression analysis was performed in all patients. As shown in Table 3, AL was related to the 25(OH)D concentration in aqueous humor. However, there was no significant relationship between the factors related to severity of glaucoma, such as mean deviation in the visual field test or RNFL thickness, and the 25(OH)D concentration in aqueous humor (Table 3). In addition, there was no statistical significance between the duration of glaucoma, type of glaucoma medications, and number of drugs and aqueous 25(OH)D concentration (Table 3). In the control group, no factors related to the aqueous 25(OH)D concentration were found (Table 3).

**Table 3:** Multivariate linear regression analysis of factors associated with 25(OH)D concentrations in aqueous humor in patients with OAG and without OAG.

Variable	Patients with OAG				Patients without OAG			
	OR (95% CI)	*p* value*	Adjusted OR (95% CI)	*p* value**	OR (95% CI)	*p* value*	Adjusted OR (95% CI)	*p* value**
Serum vitamin D concentration	1.00 (0.99–1.02)	0.60	1.00 (0.99–1.01)	0.98	0.99 (0.99–1.01)	0.83	1.00 (0.99–1.01)	0.75
Sex								
Male	1.18 (0.88–1.59)	0.26			1.26 (1.03–1.56)	0.03	1.23 (1.00–1.52)	0.05
Female	1.00				1.00		1.00	
Age (10 years)	1.01 (0.99–1.02)	0.40			0.96 (0.90–1.04)	0.31		
Average RNFL thickness	1.00 (0.93–1.09)	0.91			–	–	–	–
Visual field MD	1.01 (0.99–1.02)	0.57			–	–	–	–
IOP	1.01 (0.98–1.03)	0.60			1.03 (1.00–1.05)	0.02	1.02 (1.00–1.04)	0.06
Spherical equivalent	0.96 (0.92–1.01)	0.11			1.00 (0.9–71.02)	0.84		
Axial length	0.89 (0.83–0.96)	< 0.01	0.89 (0.82–0.96)	< 0.01	1.01 (0.96–1.07)	0.66		
Anterior chamber depth	1.08 (0.83–1.41)	0.55			1.08 (0.91–1.28)	0.36		
OAG duration (per months)	0.99 (0.97–1.01)	0.41						
Number of antiglaucoma medications								
1	1.00							
2	0.19 (0.02–1.90)	0.17						
3	2.52 (0.29–21.69)	0.40						
Alpha-agonist	2.38 (0.35–16.29)	0.38						
PG-analogs	2.23 (0.30–16.73)	0.44						
Dorzolamide/timolol fixed combination	0.22 (0.02–2.33)	0.21						
Brinzolamide/timolol fixed combination	0.77 (0.11–5.28)	0.79						
Αlpha-agonist/timolol fixed combination	6.89 (0.23–207.94)	0.27						

*OAG* open-angle glaucoma, *RNFL* retinal nerve fiber layer, *MD* mean deviation, *IOP* intraocular pressure, *CCT* central corneal thickness, *ECC* endothelial cell count, *CI* confidence interval, *OR* odds ratio, *PG* prostaglandin.

*Simple linear regression, **multiple linear regression.

## Discussion

This study confirmed that the 25(OH)D concentration in aqueous humor of patients with cataract with glaucoma was significantly lower than that in patients with cataract without glaucoma, while the presence of OAG had no significant correlation with the serum 25(OH)D concentration. The 25(OH)D concentration in aqueous humor of patients with OAG exhibited a significant correlation with AL. However, no statistical significance between the aqueous 25(OH)D concentration and glaucoma severity (RNFL thickness, MD) was detected.

The main purpose of this study was to determine whether the aqueous humor vitamin D concentration differs depending on the presence of glaucoma; further investigation was made into whether this difference was related to the duration of glaucoma, medications, or severity of glaucoma. Previous studies on the association between vitamin D and glaucoma generally investigated the correlation between serum concentrations and the incidence or progression of glaucoma. Among the previous studies, Yoo et al*.* reported that reduced serum vitamin D concentrations increased the risk for developing primary OAG^17^. However, no other study has investigated the relationship between glaucoma and aqueous vitamin D concentration. Vitamin D concentrations in aqueous humor may have a stronger correlation with glaucoma than its concentrations in serum. First, aqueous vitamin D can have a direct effect on tissues related to the pathophysiology of glaucoma, such as the ciliary epithelium, iris, and TM. Second, there is a blood–aqueous barrier in the eye, which makes it difficult for substances present in the serum to pass directly into aqueous humor; as a result, the serum concentration does not completely reflect the concentration of aqueous humor. Reviews of previous studies on 25(OH)D indicate a lack of any significant correlation between the serum and aqueous humor vitamin D concentrations. Cho et al*.* investigated differences in the concentrations of 25(OH)D in aqueous humor and serum of senile and diabetic patients with cataract^23^. Thus, this study could be used as basic reference data for studies on the relationship between glaucoma and aqueous humor vitamin D concentrations.

There are few studies on the relationship between aqueous vitamin D concentration and several eye diseases. Recently, Kim et al*.* examined the relationship between diabetic macular edema and vitamin D concentrations in aqueous humor. They reported that diabetic macular edema was associated with high concentrations of vitamin D in aqueous humor and that the aqueous vitamin D concentration might be another indicator of diabetic macular edema severity^24^. In the present study, the 25(OH)D concentration in aqueous humor of patients with cataract with OAG was significantly lower than that of control patients. However, in this study, there was no correlation between the 25(OH)D concentration in aqueous humor and indirect measures of glaucoma severity, i.e., RNFL thickness and MD values.

Several experimental studies have provided evidence of the correlation between decreased vitamin D concentrations in aqueous humor and glaucoma development. The changes occurring in TM cells, such as apoptosis, compound accumulation in extracellular matrix (ECM), and cytoskeletal disruption, are primary mechanisms involved in OAG development^25,26^. In particular, when the concentrations of oxygen radicals in aqueous humor increase, the TGF-β-SMAD3 pathway is activated in TM cells, which results in structural changes that increase the levels of ECM proteins, such as laminin, fibronectin, and myocilin. Such changes cause fibrosis of TM cells and increase the resistance of aqueous outflow, which then lead to increased IOP. However, when 1,25-(OH)_2_D_3_ is added after treating the oxygen radicals of TM cells, VDRs are activated and block the activation of the TGF-β-SMAD3 pathway, which then prevents fibrosis in TM cells^27^. Although structural changes in TM cells could be prevented when the concentrations of vitamin D in aqueous humor are high, at low concentrations, these changes in TM cells may increase the risk for glaucoma. However, to the best of our knowledge, no studies have measured vitamin D concentrations in aqueous humor of patients with glaucoma. Therefore, the findings of the present study may support this hypothesis. In addition, a previous study reported a decrease in IOP when topical 1,25-(OH)_2_D_3_ or its analog was injected in an animal experiment, and another study demonstrated an increased concentration in aqueous humor after the oral injection of vitamin D in rabbits. These findings indicate that vitamin D may be a potential candidate for developing a novel treatment for glaucoma.

In this study, the glaucoma group was 36 subjects, so the precision or power of the results may be low due to the small number of subjects. Further study with larger sample size might be required for more precision.

As all patients had cataract, it was difficult to eliminate the effect of cataract on the changes in vitamin D concentrations. In this study, the glaucoma group included only stable glaucoma patients who had no history of laser treatment or glaucoma surgery and who did not progress only with medications. If the study included patients with progressive visual field defect, or if the aqueous humor was collected from patients undergoing glaucoma surgery, the concentration of 25(OH)D could be measured lower than the results of this study. In addition, the correlation between the concentration of 25(OH)D in the aqueous humor and the MD values or RNFL thickness may have been statistically significant. Based on the results of this study, we plan to conduct a study by collecting a aqueous humor from patients undergoing glaucoma surgery for progressing glaucoma despite maximal antiglaucoma medication and measuring the 25(OH)D concentration. Furthermore, the effect of vitamin D transport on the various antiglaucoma topical medications used by patients with OAG could not be eliminated; In this study, OAG and control group showed a statistically significant difference in axial length values and the multivariate linear regression analysis revealed that there was a significant association between axial length and vitamin D concentration in aqueous humor. Previous studies demonstrated a strong relationship between vitamin D and myopia, so the potential role of longer axial length in determining lower concentrations of vitamin D in OAG group cannot be completely excluded in our study. The previous investigations that reported an association between OAG and serum vitamin D concentrations were generally large-scale studies, whereas the present study included a small number of participants. Therefore, it is challenging to make accurate judgments about serum vitamin D concentrations. If a large number of participants were included in this study, different results may have been obtained for serum vitamin D concentrations. Moreover, we did not survey patients regarding their dietary intake of vitamin D, which could have had a significant influence on the test results.

In conclusion, vitamin D concentrations in aqueous humor of patients with cataract with glaucoma were significantly lower than those in patients with cataract without glaucoma. These findings suggest that future studies should explore the use of vitamin D as a candidate therapy for glaucoma treatment.

## Methods

### Study design

This prospective study included 126 patients who underwent uneventful cataract surgery at Gyeongsang National University Hospital between January 2017 and December 2019. This study was approved by the Gyeongsang National University Hospital Institutional Review Board (2017-01-011), and adhered to the principals of the Declaration of Helsinki. Informed consents were received from all enrolled patients. The OAG group included patients who were diagnosed with primary open angle glaucoma (POAG) or normal tension glaucoma (NTG). The inclusion criteria for subjects with OAG were the characteristic visual field loss, glaucomatous optic neuropathy(having neuroretinal rim narrowing or notching, retinal nerve fiber layer defects, or disc hemorrhage and disc asymmetry between both eyes ≧0.2), and IOP > 21 mmHg. The inclusion criteria for subjects with NTG were visual field loss, glaucomatous optic neuropathy, and IOP ≦21 mmHg. Eyes with glaucomatous visual field defects were defined as (1) outside of the normal limits on the glaucoma hemifield test; (2) a pattern of standard deviation of < 5%; or (3) a cluster of 3 points with probabilities of < 5% in at least 1 hemifield on the pattern deviation map, including at least 1 point with a probability of < 1% or a cluster of 2 points with a probability of < 1%, as confirmed by at least 2 reliable visual field examinations. In these patients with glaucoma, glaucomatous visual field defects did not progress and IOP was stably controlled with antiglaucoma medications. The patients were also provided questionnaires to assess their occupation, regular daily activity (average time the patient was exposed to sunlight during the day), and use of vitamin D supplements. Of the patients who had undergone cataract surgery and ophthalmic examinations, we examined the medical records of patients who were receiving treatment for OAG.

### Exclusion criteria

Patients who had diseases related to vitamin D, such as bone metabolism disorders or hyperparathyroidism, and patients using vitamin D supplementation were excluded. In addition, we excluded patients who had other ophthalmic diseases (vitreoretinal diseases, uveitis, and anterior segment diseases), as well as patients who underwent ophthalmological surgeries or laser treatments. In addition, patients with brunescent or hypermature cataract were excluded. Finally, cases in which the test was impossible to perform because the amount of collected aqueous humor was too low were excluded from this study.

In addition, based on a previous report that the vitamin D concentration in aqueous humor could be affected by the type of cataract^23^, we targeted only senile cataract patients, excluding those with diabetic cataract or complicated cataract.

### Ophthalmic examination and cataract surgery with anterior chamber paracentesis

All procedures were conducted as described previously^23^. All participants provided complete medical histories and underwent ophthalmologic examination, including measurements of best-corrected visual acuity and IOP, as well as slit-lamp biomicroscopy and fundus examinations. Axial anterior chamber depth (ACD) and central corneal thickness (CCT) were measured using the Pentacam system (Oculus Inc., Wetzlar, Germany). Ocular biometry and axial length (AL) measurements were performed using the IOL Master system (IOL Master 500, Carl Zeiss Meditec, Germany). The number of corneal endothelial cells was determined using CellChek XL (Konan Medical, CA, USA). RNFL and macular thickness were measured using Spectralis Optical coherence tomography (Heidelberg Engineering, Heidelberg, Germany).

A single surgeon (S.J.K.) performed all cataract surgeries. After irrigation with 5% povidone and a balanced salt solution, approximately 0.15 mL of aqueous humor was collected using a 30-gauge needle through the clear cornea near the limbus. A 2.2-mm clear corneal incision was created, and two side ports were formed. A continuous curvilinear capsulorrhexis was made using a capsulorrhexis forceps. After hydrodissection, the nucleus was phacoemulsified and the residual cortex was aspirated with an Infiniti emulsifier (Alcon Laboratories, Fort Worth, TX, USA). After filling the anterior chamber with viscoelastics, a single-piece hydrophobic acrylic IOL was inserted into the capsular bag.

### Laboratory analysis

Laboratory analyses were conducted as described previously^23^. Immediately after collection, all aqueous humor samples were transferred to the laboratory, where their 25(OH)D concentrations were measured. In contrast, all serum samples were collected and stored at − 70 °C until analysis. The 25(OH)D concentrations were measured using Elecsys Vitamin D Total Kits with the Cobas e602 module (Roche Diagnostics, Mannheim, Germany), which is an electrochemiluminescence assay that includes the ruthenium-labeled vitamin D-binding protein, biotin-labeled vitamin D, and streptavidin-coated microparticles. The coefficients of variation for four concentrations (6.8, 15.0, 28.0, and 67.0 ng/ml) of intra- and interassay were 1.7–7.8% and 2.2–10.7%, respectively.

### Statistical analysis

Clinical characteristics are presented as mean and standard deviation when they were continuous variables. Categorical variables were presented as number and proportions. The characteristics of patients with cataract and OAG were compared using independent *t*-test or Fisher’s exact test (in case of categorical characteristics).

To identify factors related to the vitamin D concentration in aqueous humor, univariate linear regression analysis was performed for each variable, and significant variables were included in the multivariate linear regression model and analyzed. However, the serum vitamin D concentrations were corrected by including them in the model regardless of their significance. Regression analysis was performed in all subjects (patients with OAG and cataract. Because ACH did not satisfy the normality assumption of the regression analysis, it was converted to a natural log value for the analysis and then replaced with an index and presented as a result.

Spearman’s correlation analysis was performed to determine the correlation between the vitamin D concentration in aqueous humor and in serum, and the difference between the aqueous humor vitamin D concentration in patients with OAG and cataract was plotted.

All statistical analyses were performed using SAS version 9.4 software (SAS Institute Inc., Cary, NC, USA). A two-tailed *p-*value of < 0.05 was considered statistically significant.

### Ethical approval

All procedures performed in studies involving human participants were in accordance with the ethical standards of the Gyeongsang National University Hospital Institutional Review Board (2017-01-011), and with the 1964 Helsinki declaration and its later amendments or comparable ethical standards.

## Data Availability

The data used for analysis for this study are available from the corresponding author upon reasonable request.

## References

[1] Quigley HA, Broman AT (2006). The number of people with glaucoma worldwide in 2010 and 2020. Br. J. Ophthalmol..

[2] Quigley HA (2011). Glaucoma. Lancet.

[3] Leite MT (2009). Ascorbic acid concentration is reduced in the secondary aqueous humour of glaucomatous patients. Clin. Exp. Ophthalmol..

[4] Navajas EV (2005). Concentration of hyaluronic acid in primary open-angle glaucoma aqueous humor. Exp. Eye Res..

[5] Picht G, Welge-Luessen U, Grehn F, Lütjen-Drecoll E (2001). Transforming growth factor beta 2 levels in the aqueous humor in different types of glaucoma and the relation to filtering bleb development. Graefes Arch. Clin. Exp. Ophthalmol..

[6] Koliakos GG (2004). Endothelin-1 concentration is increased in the aqueous humour of patients with exfoliation syndrome. Br. J. Ophthalmol..

[7] DeLuca HF (2004). Overview of general physiologic features and functions of vitamin D. Am. J. Clin. Nutr..

[8] Holick MF (2007). Vitamin D deficiency. N. Engl. J. Med..

[9] Mitri J, Pittas AG (2014). Vitamin D and diabetes. Endocrinol. Metab. Clin. North Am..

[10] Rai V, Agrawal DK (2017). Role of vitamin D in cardiovascular diseases. Endocrinol. Metab. Clin. North Am..

[11] Illescas-Montes R, Melguizo-Rodríguez L, Ruiz C, Costela-Ruiz VJ (2019). Vitamin D and autoimmune diseases. Life Sci..

[12] Muscogiuri G (2017). Vitamin D and chronic diseases: The current state of the art. Arch. Toxicol..

[13] Hwang JS, Lee YP, Shin YJ (2019). Vitamin D enhances the efficacy of topical artificial tears in patients with dry eye disease. Cornea.

[14] Tang SM (2019). Vitamin D and its pathway genes in myopia: Systematic review and meta-analysis. Br. J. Ophthalmol..

[15] Layana AG (2017). Vitamin D and age-related macular degeneration. Nutrients.

[16] Luo BA, Gao F, Qin LL (2017). The association between vitamin D deficiency and diabetic retinopathy in type 2 diabetes: A meta-analysis of observational studies. Nutrients.

[17] Yoo TK, Oh E, Hong S (2014). Is vitamin D status associated with open-angle glaucoma? A cross-sectional study from South Korea. Public Health Nutr..

[18] Goncalves A (2015). Serum vitamin D status is associated with the presence but not the severity of primary open angle glaucoma. Maturitas.

[19] Vuković Arar Ž (2016). Association between serum vitamin D level and glaucoma in women. Acta Clin. Croat..

[20] Lv Y (2016). Associations of vitamin D deficiency and vitamin D receptor (Cdx-2, Fok I, Bsm I and Taq I) polymorphisms with the risk of primary open-angle glaucoma. BMC Ophthalmol..

[21] Kim HT (2016). The relationship between vitamin D and glaucoma: A Kangbuk Samsung health study. Korean J. Ophthalmol..

[22] Li S (2017). Lack of association between serum vitamin B_6_, vitamin B_12_, and vitamin D levels with different types of glaucoma: A systematic review and meta-analysis. Nutrients.

[23] Cho MC (2020). Aqueous humor and serum 25-hydroxyvitamin D levels in patients with cataracts. BMC Ophthalmol..

[24] Kim KL (2019). Serum and aqueous humor vitamin D levels in patients with diabetic macular edema. Graefes Arch. Clin. Exp. Ophthalmol..

[25] Tamm ER, Braunger BM, Fuchshofer R (2015). Intraocular pressure and the mechanisms involved in resistance of the aqueous humor flow in the trabecular meshwork outflow pathways. Prog. Mol. Biol. Transl. Sci..

[26] Awai-Kasaoka N (2013). Oxidative stress response signaling pathways in trabecular meshwork cells and their effects on cell viability. Mol. Vis..

[27] Lv Y (2019). 1α,25-Dihydroxyvitamin D3 attenuates oxidative stress-induced damage in human trabecular meshwork cells by inhibiting TGFβ-SMAD3-VDR pathway. Biochem. Biophys. Res. Commun..

